# Alcohol-Related Perceptions and High-Risk Drinking Among Brazilian College Students: The Role of Social Contexts, Motives, and Risk Behaviors

**DOI:** 10.3390/healthcare14131993

**Published:** 2026-07-04

**Authors:** Denise De Micheli, Geisa Gomes da Silva, Richard Alecsander Reichert, Adriana Scatena, Laura Soares da Silva, Vinícius de Souza Marchesano, Bruno Barros Dutra, Evelin Moreira Freires, Amanda Severo Lins Vitta, Wanderlei Abadio de Oliveira, Claudio Romualdo, Makilim Nunes Baptista, André Luiz Monezi Andrade

**Affiliations:** 1 Department of Psychobiology, Universidade Federal de São Paulo (UNIFESP), Rua Botucatu, 862, Vila Clementino, Sao Paulo 04023-062, SP, Brazil; demicheli.unifesp@gmail.com (D.D.M.); geisa.gomes@unifesp.br (G.G.d.S.); reichert@unifesp.br (R.A.R.); 2 Psychology Section, Santo André Medical Center, R. Cel. Fernando Prestes, 78, Centro, Santo André 09020-110, SP, Brazil; adrianascatena77@gmail.com; 3 School of Life Sciences, Pontifical Catholic University of Campinas (PUC-Campinas), Av. John Boyd Dunlop, S/N, Jardim Ipaussurama, Campinas 13034-685, SP, Brazil; laursoaresilv@hotmail.com (L.S.d.S.); vinicius.march.acd@gmail.com (V.d.S.M.); bruno.bd2@puccampinas.edu.br (B.B.D.); evelin.mfreires@gmail.com (E.M.F.); severolins.amanda@gmail.com (A.S.L.V.); wanderlei.oliveira@puc-campinas.edu.br (W.A.d.O.); claudio.romualdo42@gmail.com (C.R.); makilim.nunes@puc-campinas.edu.br (M.N.B.)

**Keywords:** alcohol use, high-risk drinking, alcohol-related perceptions, alcohol expectancies, drinking motives, college students

## Abstract

**Highlights:**

**What are the main findings?**

- Positive alcohol-related perceptions were associated with higher odds of high-risk alcohol use, whereas negative perceptions were inversely associated with risk indicators.
- Drinking in nightclubs/parties and bars, and driving after drinking, were strongly associated with high-risk alcohol use among Brazilian college students.

**What are the implications of the main findings?**

- University-based alcohol prevention efforts may benefit from addressing the perceived social and emotional rewards of drinking, not only alcohol-related harms.
- These findings highlight the relevance of high-risk social settings, driving after drinking, and combined alcohol use with other substances as potential targets for future harm-reduction research and intervention planning.

**Abstract:**

**Background/Objectives**: Alcohol consumption is a major public health concern among university students. However, less is known about how positive and negative alcohol-related perceptions are jointly associated with drinking contexts, motives, and risk behaviors in Brazilian college samples. This study examined positive and negative perceptions of alcohol use and their associations with high-risk drinking, drinking contexts, motivations, and risk behaviors among Brazilian college students. **Methods**: This cross-sectional online study included 367 Brazilian college students aged 18 to 30 years (M = 22.2; SD = 2.92). Alcohol use was assessed using the AUDIT-C, and perceptions were measured using the Scale of Perceptions about Alcohol Consumption in Higher Education Students. Analyses included descriptive statistics, Mann–Whitney U tests, chi-square tests, Spearman correlations, and age- and gender-adjusted logistic regression models. **Results**: High-risk alcohol use was identified in 55.6% of participants. Positive perceptions were associated with higher odds of high-risk alcohol use (aOR = 4.85), whereas negative perceptions were associated with lower odds (aOR = 0.50). Drinking in nightclubs/parties (aOR = 4.44) and bars (aOR = 3.91) was associated with higher odds of high-risk alcohol use. Driving after drinking showed the strongest association among risk behaviors (aOR = 5.25), and marijuana was the only other substance significantly associated with this outcome. **Conclusions**: High-risk drinking among Brazilian college students was associated with perceived alcohol benefits, social drinking contexts, drinking motives, and selected risk behaviors. Prevention strategies should address the perceived social and emotional rewards of drinking. Findings should be interpreted as associations within this cross-sectional convenience sample.

## 1. Introduction

Alcohol consumption is a major global public health challenge with biological, psychological, and social implications. It is linked to behavioral changes, dependence, and physical and socioeconomic harms [1], affecting personal, academic, and social life [2,3]. The World Health Organization recognizes alcohol use as a major contributor to the global burden of disease, including noncommunicable diseases such as cancer and cardiovascular diseases, as well as injuries from traffic accidents and violence [4].

Early alcohol use strongly predicts later alcohol-related problems, including dependence [5]. During the transition to adulthood, binge drinking, commonly defined as consuming 4–5 drinks on a single occasion, is especially prevalent [6]. Among university students, high-risk drinking patterns are often linked to academic stress, emotional instability, and psychosocial vulnerabilities [7,8].

Cognitive models and Alcohol Expectancy Theory suggest that drinking behavior may be shaped by beliefs about the anticipated effects of alcohol use [9,10]. Positive expectations, such as pleasure, stress relief, and increased sociability, may increase the likelihood of alcohol use. Conversely, negative expectations, including cognitive impairment and aggressive behavior, may discourage alcohol use, though they may be insufficient to inhibit drinking in permissive social settings [9,10]. Complementarily, Social Learning Theory highlights the role of peer modeling, reinforcement, and observational learning in maintaining substance use despite recognized harms [11]. In the present study, perceptions refer to students’ self-reported evaluations of the positive and negative effects of alcohol use, whereas alcohol expectancies refer to the broader theoretical framework for anticipated outcomes of drinking.

Primary motivations for consumption include enjoyment, curiosity, and socialization [12]. However, high-risk consumption patterns are linked to significant physical and socioeconomic harms [1]. Studies consistently link heavy drinking to risk behaviors, including interpersonal violence [13], physical or sexual assault, and unprotected sexual activity [14,15]. Furthermore, high-risk alcohol use has been associated with adverse outcomes such as health problems, sleep disturbances, interpersonal difficulties, and decreased academic performance [15]. Recent evidence indicates that alcohol-related expectations may begin to develop in early adolescence. Parental monitoring and clear rules about drinking may help shape these expectations before university entry, particularly when reinforced by peer disapproval [16].

International research from Portugal [17], the United States [14], and Australia [18] highlights that students often overestimate peers’ substance use and face social pressure to drink excessively. In diverse cultural settings, including India [19] and Saudi Arabia [20], social norms and beliefs that frame alcohol as a means of social integration or emotional relief have been linked to continued alcohol use [21,22].

Although alcohol expectancies and drinking motives have been widely examined in international research, fewer studies have jointly examined positive and negative perceptions of alcohol use, drinking contexts, motivations, and alcohol-related risk behaviors among Brazilian college students. This gap is significant because university students’ alcohol use is influenced not just by personal beliefs, but also by cultural and social drinking environments, peer interactions, and the perceived benefits of drinking. In Brazil, where university drinking takes place in diverse social settings, analyzing these factors together can shed light on which perceptions and contexts most strongly connect to high-risk drinking.

This study aimed to examine positive and negative perceptions of alcohol use and their associations with high-risk drinking, drinking contexts, motivations, and alcohol-related risk behaviors among Brazilian college students. Drawing on Alcohol Expectancy Theory and Social Learning Theory, we hypothesized that high-risk drinkers would endorse stronger positive perceptions of alcohol use than low-risk drinkers, whereas low-risk drinkers would more strongly endorse negative perceptions. We also expected high-risk alcohol use to be associated with social drinking settings, particularly bars and nightclubs/parties. Finally, we hypothesized that high-risk alcohol use would be associated with drinking motives related to enjoyment, peer influence, and relaxation, as well as selected risk behaviors, including driving after drinking, sexual intercourse under the influence of alcohol, and alcohol use combined with other substances.

## 2. Materials and Methods

### 2.1. Study Design and Sample

This cross-sectional observational study used a convenience sample of Brazilian college students recruited via an online survey. The study was reported in accordance with the STROBE guidelines for cross-sectional studies.

Participants were recruited through an online survey link distributed via social media and by instructors at higher education institutions. Because the link was shared through open online channels, it was not possible to determine the total number of students who received or accessed the invitation; therefore, a formal response rate could not be calculated. Inclusion criteria were being 18 years of age or older, being actively enrolled in higher education, and providing informed consent to participate in the study. A total of 374 responses were initially recorded. Seven responses were excluded: two from participants younger than 18 years and five due to incomplete or inadequate completion of the study instruments. Therefore, the final analytic sample included 367 Brazilian college students aged 18 to 30 years, with a mean age of 22.2 years (SD = 2.92). Most participants were female (64.6%), followed by male (33.2%), non-binary (1.6%), and gender-fluid students (0.5%).

### 2.2. Instruments

A structured questionnaire developed for this study was used to collect sociodemographic information and alcohol-related contextual variables. It included 21 items assessing age, gender, age at first alcohol use, first and current drinking contexts, usual drinking companions, driving after drinking, riding with an intoxicated driver, and main reasons for drinking. Because this questionnaire was used solely to characterize contextual and behavioral variables and was not intended as a psychometric scale, internal consistency coefficients were not calculated.

Perceptions of alcohol consumption, both positive and negative, were assessed using the Scale of Perceptions about Alcohol Consumption in Higher Education Students [17]. The instrument comprises 10 items across two dimensions, positive and negative perceptions, rated on a 6-point Likert scale from 1 (“strongly disagree”) to 6 (“strongly agree”), with higher scores indicating stronger agreement with each perception. Example items for positive perceptions include “Using alcohol increases enjoyment” and “Using alcohol allows one to pass the time better,” while negative perceptions include “Using alcohol creates dependence and leads to increased consumption” and “Using alcohol leads to aggressive and unsociable behaviors”. For analysis, responses were sorted into three groups: disagree, partially agree, and agree. This kept the original order of the answers and made them easier to understand. In this sample, internal consistency was good for positive perceptions (α = 0.861; ω = 0.862) and negative perceptions (α = 0.825; ω = 0.827).

Alcohol consumption was assessed using the AUDIT-C (Alcohol Use Disorders Identification Test—Concise), a three-item screening instrument validated for the Brazilian population. The items assess the frequency of alcohol use, the typical number of drinks consumed, and the frequency of consuming six or more drinks on a single occasion over the past three months. The total AUDIT-C score ranges from 0 to 12. For analytical purposes, scores ≥ 4 for women and ≥ 5 for men were classified as high-risk alcohol use, whereas scores below these cutoffs were classified as low-risk alcohol use. This classification was used for group comparisons and as a dependent variable in logistic regression models. For participants who identified as non-binary or gender fluid, the same AUDIT-C cutoff used for women was applied as a cautious screening measure, since there are no validated AUDIT-C thresholds for these genders in Brazil. Although these participants were included in the overall analyses, they were not examined separately in gender-specific comparisons because of the small sample size.

### 2.3. Data Analysis

Descriptive analyses included absolute frequencies, percentages, means, and standard deviations. A sensitivity analysis based on the final sample of 367 participants, with α = 0.05 and 80% power, showed that the sample size was adequate to detect small-to-moderate associations. Specifically, it could identify roughly Cramér’s V = 0.16 in 2 × 3 chi-square tests and Spearman’s rho = 0.15 in correlation analyses. Normality of continuous and ordinal variables was assessed with the Shapiro–Wilk test. Because the perception items were ordinal and did not meet normality assumptions, differences between low- and high-risk alcohol use groups were examined using the Mann–Whitney U test. This test was applied to compare positive and negative alcohol-related perception items according to AUDIT-C risk classification. Associations between alcohol-risk groups and categorical variables, including drinking locations, reasons for drinking, risk behaviors, and alcohol use combined with other substances, were evaluated with chi-square tests.

Binomial logistic regression models estimated associations between these variables and two outcomes: high-risk alcohol use and frequent consumption of six or more drinks. The primary regression models were adjusted for age and gender. To assess the potential impact of additional confounders, sensitivity analyses were conducted using expanded logistic regression models that were adjusted for age, gender, age at first alcohol use, family alcohol-related problems, type of university, study load, and field of study.

As a complementary analysis, Spearman correlations were computed to examine associations between positive and negative perception scores, the AUDIT-C total score, and alcohol consumption indicators, including frequency of alcohol use, typical number of drinks, frequency of consuming six or more drinks, and age at first alcohol use. Only complete responses were included in the analyses; therefore, no imputation was applied. All analyses were conducted in Jamovi (version 2.7.15), with significance set at 5%.

## 3. Results

### 3.1. Sociodemographic Characteristics and Alcohol Consumption Profile

Table 1 presents the sociodemographic characteristics and alcohol consumption profile of the sample. Regarding alcohol consumption, most participants reported regular use (81.5%), with initiation occurring mainly during adolescence, especially between 11 and 15 years and between 16 and 20 years. More than half of the sample reported family problems related to alcohol use and were classified as having high-risk alcohol use according to the AUDIT-C. When AUDIT-C classification was examined within gender groups, high-risk alcohol use was observed in 57.4% of female participants and 52.5% of male participants. Non-binary and gender-fluid participants were included in the overall gender distribution but were not analyzed separately in the gender-stratified AUDIT-C classification due to the small sample size.

### 3.2. Positive and Negative Perceptions Regarding Alcohol Consumption

Table 2 presents the distribution of responses to the positive and negative perception items and their associations with AUDIT-C risk classification. Most perception items differed significantly between low- and high-risk drinkers, except for the perception that alcohol causes memory lapses or loss of awareness. The strongest associations were observed for positive perceptions, particularly enjoyment, stress relief, disinhibition, and passing time. Among negative perceptions, the strongest associations were with academic impairment, public disturbance or problems with the police, aggressive or antisocial behavior, and dependence.

### 3.3. Differences in Perceptions Regarding Alcohol Consumption

**Figure 1:**
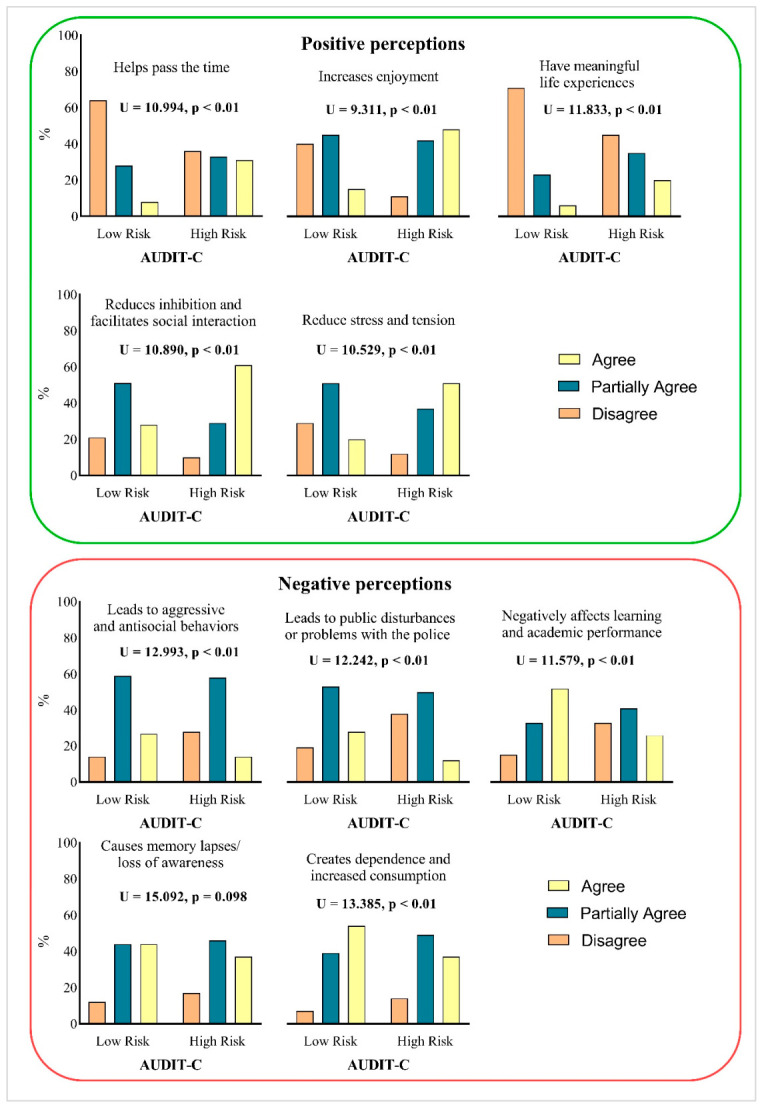
Positive and negative perceptions of alcohol use among low- and high-risk drinkers according to AUDIT-C classification. Bars represent percentages within each AUDIT-C risk group. Group differences were tested using the Mann–Whitney U test. AUDIT-C = Alcohol Use Disorders Identification Test—Concise.

Overall, high-risk drinkers endorsed more positive perceptions of alcohol use, particularly those related to passing time, meaningful experiences, enjoyment, disinhibition, and stress relief (Figure 1). In contrast, low-risk drinkers more strongly endorsed negative perceptions, especially those related to aggressive or antisocial behavior, public disturbance, or problems with the police, academic impairment, and dependence. The perception that alcohol causes memory lapses or loss of awareness did not differ significantly between groups.

### 3.4. Alcohol-Related Perceptions, Drinking Locations, and Risky Consumption

In age- and gender-adjusted logistic regression models, positive perceptions were associated with higher odds of high-risk alcohol use, whereas negative perceptions were associated with lower odds (Table 3). Drinking in bars, nightclubs/parties, and at home was also associated with higher odds of high-risk alcohol use, with the strongest association observed for nightclubs/parties. For frequent consumption of six or more drinks, drinking in bars was associated with higher odds, whereas drinking at home was associated with lower odds.

### 3.5. Complementary Correlations Among Perceptions and Alcohol Consumption Indicators

**Figure 2:**
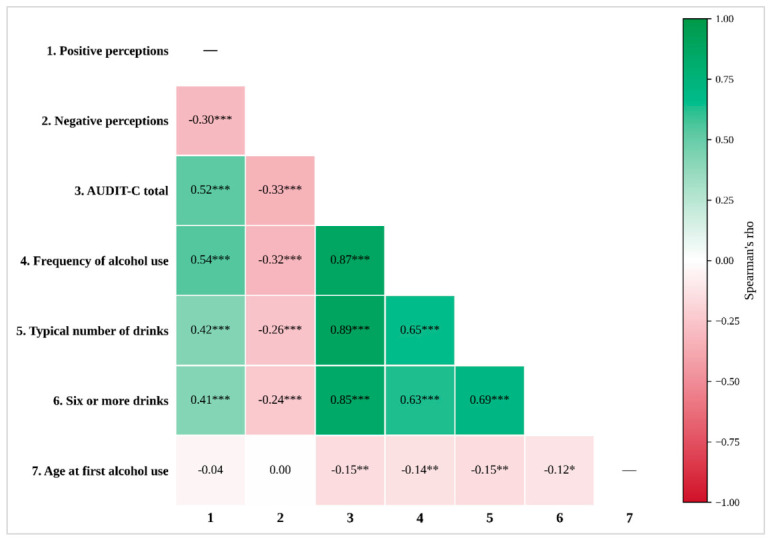
Spearman correlations among alcohol-related perceptions, AUDIT-C scores, and alcohol consumption indicators. Note: Values represent Spearman’s rho coefficients. Dashes indicate non-reported duplicated correlations or self-correlations. Positive and negative perception scores were calculated as the mean of the five items in each dimension. Age at first alcohol use was coded from earlier to later initiation. * *p* < 0.05; ** *p* < 0.01; *** *p* < 0.001.

Regarding the correlations (Figure 2), positive perceptions were positively correlated with AUDIT-C total scores, more frequent alcohol use, typical drinking amounts, and regular consumption of six or more drinks. Conversely, negative perceptions were inversely correlated with these measures of alcohol consumption. Additionally, earlier alcohol initiation showed a weak association with higher AUDIT-C scores and more substantial drinking patterns.

### 3.6. Risk Behaviors, Motivations, and Alcohol Consumption

High-risk alcohol consumption was associated with sexual intercourse under the influence of alcohol, driving after drinking, and combining alcohol with other substances (Table 4). In contrast, involvement in fights, riding with a drunk driver, and unprotected sex were not significantly associated with high-risk alcohol use in the adjusted models. Driving after drinking showed the strongest association with high-risk alcohol use. Regarding drinking motives, enjoyment, peer influence (“everyone drinks”), relaxation, and socializing were associated with higher odds of high-risk alcohol use in the adjusted models. Curiosity, drinking to forget problems, enjoying drinking, and loss of inhibitions were not significantly associated with high-risk alcohol use. Among other substances, marijuana was the only substance significantly associated with high-risk alcohol use in the adjusted model. Tobacco, electronic cigarettes, hookah, inhalants, cocaine/crack, and psychedelics were not significantly associated with high-risk alcohol use.

### 3.7. Sensitivity Analyses

Sensitivity analyses with expanded adjustment models were performed to test the robustness of the primary results, shown in Appendix A (Table A1 and Table A2). The core associations largely remained consistent after controlling for additional covariates. However, the link between sexual intercourse under the influence of alcohol lost statistical significance, and some low-frequency substance-use variables had wide confidence intervals, suggesting these secondary findings should be interpreted with caution.

## 4. Discussion

This study examined positive and negative perceptions of alcohol use and their associations with high-risk drinking, drinking contexts, motivations, and risk behaviors among Brazilian college students. Overall, the findings were consistent with the main hypotheses. High-risk drinkers endorsed stronger positive perceptions of alcohol use, whereas low-risk drinkers more strongly endorsed negative perceptions. In adjusted models, positive perceptions were associated with higher odds of high-risk alcohol use, while negative perceptions were associated with lower odds. Complementary Spearman correlations reinforced this pattern, showing that positive perceptions were positively correlated with AUDIT-C total scores and indicators of heavier drinking, whereas negative perceptions were inversely correlated with these variables. These findings suggest that high-risk drinking in this sample was associated not only with individual perceptions of alcohol but also with social contexts, motivations, and risk-related behaviors.

The differences between low- and high-risk drinkers align with Alcohol Expectancy Theory, which posits that anticipated positive outcomes of drinking may increase the likelihood of alcohol use, whereas negative expectations may discourage consumption. Prior research indicates that positive expectancies are more common among individuals with earlier alcohol exposure and may contribute to more stable beliefs about alcohol over time [9,10]. In the present study, high-risk students placed greater weight on positive perceptions, including disinhibition, enjoyment, and relaxation. In contrast, low-risk students emphasized negative consequences, such as dependence, aggressive behavior, and academic impairment. This pattern is consistent with evidence that positive expectations are associated with heavier drinking, whereas negative perceptions may serve as protective beliefs in some contexts [17,23,24]. The correlation analysis supported this interpretation by showing that positive perceptions were associated with higher AUDIT-C scores and heavier drinking indicators, whereas negative perceptions were associated with the opposite.

The association between high-risk drinking and social settings, especially bars and nightclubs/parties, also supports the relevance of Social Learning Theory for understanding alcohol use in university contexts. These environments may increase exposure to peer modeling, social reinforcement, and permissive drinking norms. Previous studies have similarly shown that socially stimulating drinking contexts are associated with stronger positive alcohol expectancies and heavier drinking patterns among college students [25,26]. In this study, drinking at home showed a different pattern: it was associated with high-risk alcohol classification but with lower odds of frequent consumption of six or more drinks, suggesting that drinking location may relate differently to general risk classification and heavier episodic intake.

Drinking motives further helped characterize high-risk alcohol use in this sample. Enjoyment, peer influence, relaxation, and socializing were associated with high-risk drinking, suggesting that enhancement, social, and coping-related motives may be relevant among Brazilian college students. This finding aligns with evidence that social motives are among the most common reasons for drinking among young adults, followed by emotional and coping motives [27,28]. Although alcohol may be perceived as a short-term strategy for relaxation or stress relief, drinking to cope has been linked to poorer mental health indicators, including depression, anxiety, loneliness, and stress [29]. This reinforces the need to consider not only drinking frequency but also the subjective reasons students report for drinking.

High-risk alcohol use was also associated with risk behaviors, including driving after drinking, sexual intercourse under the influence of alcohol, and combining alcohol with other substances. Driving after drinking showed the strongest association, which is particularly concerning given its potential legal and physical consequences. Among the substances assessed, marijuana was the only one significantly associated with high-risk alcohol use in the adjusted model. These findings suggest that high-risk drinking may co-occur with other risk behaviors, underscoring the importance of prevention strategies that address alcohol use within broader behavioral and social contexts rather than as isolated behavior.

These findings have practical implications for prevention in university settings. Because this was a cross-sectional study, these implications should be interpreted as hypotheses for prevention planning rather than evidence of causal effects. Interventions should not focus solely on informing students about the harm of alcohol, since many students appear to recognize negative consequences while still endorsing positive social and emotional effects. Prevention programs may benefit from addressing the perceived benefits of drinking, challenging normative beliefs about peer alcohol use, and questioning the idea that alcohol is necessary for enjoyment, relaxation, or social connection. Harm-reduction strategies may also consider high-risk contexts such as bars and parties. Specific attention may be warranted for driving after drinking and for the combined use of alcohol with other substances.

The sensitivity analyses further confirmed the robustness of the main findings, though some associations should be interpreted with caution. After additionally adjusting for factors such as age at first alcohol use, family alcohol-related problems, university type, study load, and field of study, the results related to positive perceptions, drinking in bars and parties, driving after drinking, alcohol combined with other drugs, enjoyment, peer influence, relaxation, socializing, and marijuana use remained largely unchanged. However, the association with sexual intercourse under the influence of alcohol was no longer statistically significant, and some low-frequency substance-use variables had wide confidence intervals. These results indicate that the most consistent and reliable findings are distinguished from less precise or secondary estimates.

This study has limitations. Its cross-sectional design precludes causal inference, and the observed associations should not be interpreted as indicating directionality between alcohol-related perceptions, drinking contexts, motives, risk behaviors, and high-risk alcohol use. The convenience sample collected online also limits the generalizability of the findings to all Brazilian college students, particularly because students with a greater interest in alcohol-related topics or stronger engagement with online recruitment channels may have been more likely to participate. Self-reported measures may have introduced recall or social desirability bias. Although sensitivity analyses with expanded covariate adjustment were conducted, residual confounding remains possible because not all potentially relevant variables were available or included. In addition, some substance-use variables had low frequencies, resulting in wide confidence intervals and reduced precision for these estimates. Therefore, findings involving low-frequency substances should be considered exploratory. The correlation analyses should also be interpreted as complementary and non-causal. Future studies should use longitudinal and multicenter designs, include more diverse student populations, and examine whether positive alcohol perceptions mediate or moderate the association between social contexts and high-risk drinking.

## 5. Conclusions

This study showed that high-risk alcohol use among Brazilian college students was associated with stronger positive perceptions of alcohol, social drinking contexts, drinking motives, and selected risk behaviors. Negative perceptions, in contrast, were more strongly endorsed by low-risk drinkers and were inversely associated with AUDIT-C scores and alcohol consumption indicators. These findings indicate that university alcohol research and prevention efforts might be more effective if they focus not only on understanding alcohol-related harms but also on the social and emotional rewards that may encourage high-risk drinking. Since the study used a cross-sectional, convenience sample, these results should be viewed as associations specific to this sample and need validation through longitudinal and multicenter research.

## Figures and Tables

**Table 1:** Sociodemographic data of participants (*n* = 367).

Category	Subcategory	*N*	%
Gender	Female	237	64.6
	Male	122	33.2
	Non-binary	6	1.6
	Gender fluid	2	0.5
Education system	Public	208	56.7
	Private	143	39.0
	Cooperative	16	4.4
Study load	Part time	262	71.4
	Full time	105	28.6
Field of study	Humanities	223	60.8
	Biological/Health Sciences	83	22.6
	Exact Sciences	61	16.6
Alcohol consumption	Regular users	299	81.5
	Non-users	68	18.5
Age of onset of alcohol consumption	0–5 years	5	1.4
	6–10 years	17	4.6
	11–15 years	147	40.1
	16–20 years	185	50.4
	21–25 years	10	2.7
	26–30 years	3	0.8
Problems with alcohol use in the family	Yes	226	61.6
	No	78	21.3
	Unknown	63	17.2
AUDIT-C classification	High risk	204	55.6
	Low risk	163	44.4
AUDIT-C classification by gender	Female: high risk	136	57.4
	Female: low risk	101	42.6
	Male: high risk	64	52.5
	Male: low risk	58	47.5
	Non-binary: high risk	3	50.0
	Non-binary: low risk	3	50.0
	Gender fluid: high risk	1	50.0
	Gender fluid: low risk	1	50.0

Note: Percentages are based on the total sample, except for AUDIT-C classification by gender, which is presented as percentages within each gender category. AUDIT-C = Alcohol Use Disorders Identification Test—Concise.

**Table 2:** Data on positive and negative perceptions of alcohol use (*n* = 367). Data expressed as percentages.

Item Evaluated	Agree *n* (%)	Partially Agree *n* (%)	Disagree *n* (%)	χ^2^	*p*	Effect
**Specific negative perceptions**						
Drinking alcohol leads to aggressive and antisocial behavior	72 (19.6)	214 (58.3)	81 (22.1)	16.57	***	0.21
Drinking alcohol is addictive and leads to increased consumption	163 (44.4)	164 (44.7)	40 (10.9)	12.62	**	0.19
Drinking alcohol causes disturbances of peace or problems with the police	71 (19.3)	188 (51.2)	108 (29.4)	22.87	***	0.25
Drinking alcohol causes memory lapses/loss of awareness	147 (40.1)	165 (45.0)	55 (15.0)	2.81	0.24	0.09
Drinking alcohol negatively affects learning and academic performance	137 (37.3)	137 (37.3)	93 (25.3)	28.81	***	0.28
**Specific positive perceptions**						
Drinking alcohol increases enjoyment	122 (33.2)	158 (43.1)	87 (23.7)	60.83	***	0.41
Drinking alcohol makes it easier to pass the time	75 (20.4)	113 (30.8)	179 (48.8)	37.96	***	0.32
Drinking alcohol facilitates meaningful life experiences	50 (13.6)	109 (29.7)	208 (56.7)	29.02	***	0.28
Drinking alcohol helps relieve stress or tension	137 (37.3)	158 (43.1)	72 (19.6)	43.26	***	0.34
Drinking alcohol loosens inhibitions and facilitates contact with colleagues	169 (46.0)	143 (39.0)	55 (15.0)	40.65	***	0.33

Note: Percentages are based on the total sample. Chi-square tests compared the distribution of responses across AUDIT-C low-risk and high-risk groups. Effect = Cramér’s V indicates the magnitude of association. AUDIT-C = Alcohol Use Disorders Identification Test—Concise. ** *p* < 0.01; *** *p* < 0.001.

**Table 3:** Age- and gender-adjusted associations of alcohol-related perceptions and drinking locations with high-risk alcohol use and frequent consumption of six or more drinks.

Variables Examined	High-Risk Alcohol Use		
	aOR	95% CI	*p*
**Alcohol-related perceptions**			
Positive perceptions	4.85	3.05–7.69	***
Negative perceptions	0.50	0.31–0.79	**
**Drinking locations**			
Bars	3.91	2.33–6.55	***
Restaurants	0.64	0.32–1.28	0.21
Snack bars	1.45	0.52–4.03	0.48
Nightclubs/parties	4.44	2.68–7.36	***
At home	1.95	1.16–3.27	*
**Frequent consumption of six or more drinks**			
Drinking locations			
Bars	2.16	1.29–3.63	**
Restaurants	0.99	0.53–1.86	0.97
Snack bars	0.74	0.28–1.99	0.56
Nightclubs/parties	0.66	0.41–1.08	0.10
At home	0.42	0.26–0.70	***

Note: Logistic regression models were adjusted for age and gender. Positive and negative perception scores were entered simultaneously in the perception model. For drinking locations, the reference category was “No”. aOR = adjusted odds ratio; CI = confidence interval. * *p* < 0.05; ** *p* < 0.01; *** *p* < 0.001.

**Table 4:** Age- and gender-adjusted associations between alcohol-related factors and high-risk alcohol use.

Alcohol-Related Factors	Risk Behaviors and Consequences		
	aOR	95% CI	***p***
Sexual intercourse under the influence of alcohol	1.91	1.01–3.62	*
Fights	0.50	0.23–1.09	0.08
Driving after drinking	5.25	2.37–11.63	***
Riding with a drunk driver	1.26	0.74–2.16	0.39
Unprotected sex	1.35	0.69–2.65	0.38
Combined alcohol with other drugs	2.76	1.60–4.76	***
	**Drinking motives**		
	**aOR**	**95% CI**	***p***
Enjoyment	3.60	2.17–5.97	***
Curiosity	1.29	0.74–2.23	0.37
Peer influence/everyone drinks	2.77	1.30–5.90	**
Forgetting problems	2.73	0.98–7.62	0.05
Relaxation	1.96	1.12–3.45	*
Enjoy drinking	1.61	0.90–2.87	0.11
Socializing	1.96	1.03–3.75	*
Loss of inhibitions	1.60	0.64–4.05	0.32
	**Other substance use**		
	**aOR**	**95% CI**	***p***
Tobacco	1.26	0.67–2.38	0.47
Electronic cigarette	5.10	1.00–26.05	0.05
Hookah	0.66	0.03–15.61	0.80
Inhalants	3.14	0.31–32.37	0.34
Marijuana	2.47	1.41–4.33	**
Cocaine/crack	1.93	0.48–7.81	0.36
Psychedelics	2.25	0.70–7.23	0.17

Note: Separate logistic regression models were estimated for each alcohol-related factor. Models were adjusted for age and gender. For all variables, the reference category was “No”. aOR = adjusted odds ratio; CI = confidence interval. * *p* < 0.05; ** *p* < 0.01; *** *p* < 0.001.

## Data Availability

The data are not publicly available due to privacy and ethical restrictions.

## Appendix A. Sensitivity Analyses

The sensitivity analyses were conducted using expanded logistic regression models adjusted for age, gender, age at first alcohol use, family alcohol-related problems, type of university, study load, and field of study. These analyses were performed to assess whether the main associations observed in the primary models remained robust after additional covariate adjustment. Overall, the main findings were generally consistent. Positive perceptions, drinking in bars and nightclubs/parties, driving after drinking, combined alcohol use with other drugs, enjoyment, peer influence, relaxation, socializing, and marijuana use remained associated with high-risk alcohol use. However, sexual intercourse under the influence of alcohol was no longer statistically significant, and some low-frequency substance-use variables, including electronic cigarette use, hookah, inhalants, cocaine/crack, and psychedelics, showed wide confidence intervals, suggesting imprecision and the need for cautious interpretation.

**Table A1:** Sensitivity analysis using expanded adjustment models for associations of alcohol-related perceptions and drinking locations with high-risk alcohol use and frequent consumption of six or more drinks.

Variables Examined	Outcome	aOR	95% CI	*p*
**Alcohol-related perceptions**				
Positive perceptions	High-risk alcohol use	4.76	2.99–7.58	***
Negative perceptions		0.42	0.25–0.70	***
**Drinking locations**				
Bars	High-risk alcohol use	4.24	2.47–7.27	***
Restaurants		0.64	0.32–1.29	0.21
Snack bars		1.41	0.50–4.01	0.52
Nightclubs/parties		4.43	2.62–7.49	***
At home		2.10	1.23–3.57	**
**Drinking locations**				
Bars	Frequent consumption of six or more drinks	9.39	4.25–20.71	***
Restaurants		1.43	0.68–3.01	0.35
Snack bars		0.77	0.27–2.22	0.63
Nightclubs/parties		1.77	0.93–3.37	0.08
At home		0.59	0.31–1.10	0.10

Note: Sensitivity analyses used expanded logistic regression models adjusted for age, gender, age at first alcohol use, family alcohol-related problems, type of university, study load, and field of study. Positive and negative perception scores were entered simultaneously in the perception model. For drinking locations, the reference category was “No”. aOR = adjusted odds ratio; CI = confidence interval. ** *p* < 0.01; *** *p* < 0.001.

**Table A2:** Sensitivity analysis using expanded adjustment models for associations between alcohol-related factors and high-risk alcohol use.

Alcohol-Related Factors	aOR	95% CI	*p*
**Risk behaviors and consequences**			
Sexual intercourse under the influence of alcohol	1.87	0.97–3.60	0.06
Fights	0.53	0.25–1.16	0.11
Driving after drinking	4.77	2.08–10.95	***
Riding with a drunk driver	1.20	0.69–2.08	0.51
Unprotected sex	1.31	0.66–2.60	0.44
Combined alcohol with other drugs	3.00	1.71–5.26	***
**Drinking motives**			
Enjoyment	3.47	2.06–5.85	***
Curiosity	1.43	0.81–2.50	0.22
Peer influence/everyone drinks	2.86	1.30–6.30	**
Forgetting problems	2.93	1.05–8.17	*
Relaxation	2.06	1.16–3.66	*
Enjoy drinking	1.53	0.84–2.79	0.17
Socializing	2.04	1.05–3.96	*
Loss of inhibitions	1.38	0.53–3.60	0.50
**Other substance use**			
Tobacco	1.37	0.72–2.60	0.34
Electronic cigarette	5.36	1.01–28.39	*
Hookah	0.66	0.02–18.62	0.81
Inhalants	2.59	0.24–28.13	0.43
Marijuana	2.61	1.46–4.67	**
Cocaine/crack	1.62	0.39–6.78	0.51
Psychedelics	2.26	0.70–7.34	0.17

Note: Sensitivity analyses used expanded logistic regression models adjusted for age, gender, age at first alcohol use, family alcohol-related problems, type of university, study load, and field of study. For all alcohol-related factors, the reference category was “No”. aOR = adjusted odds ratio; CI = confidence interval. * *p* < 0.05; ** *p* < 0.01; *** *p* < 0.001.
